# For a proper use of frequentist inferential statistics in public health

**DOI:** 10.1016/j.gloepi.2024.100151

**Published:** 2024-06-15

**Authors:** Alessandro Rovetta, Mohammad Ali Mansournia, Alessandro Vitale

**Affiliations:** a Research & Disclosure, R&C Research, Bovezzo (BS), Italy; b Department of Epidemiology and Biostatistics, School of Public Health, Tehran University of Medical Sciences, Tehran, Iran; c Department of Surgical, Oncological and Gastroenterological Sciences (DiSCOG), Padova University, Padova, Italy

**Keywords:** Clinical significance, Confidence intervals, Null hypothesis, Nullism, Statistical compatibility, Statistical significance

## Abstract

As widely noted in the literature and by international bodies such as the American Statistical Association, severe misinterpretations of *P*-values, confidence intervals, and statistical significance are sadly common in public health. This scenario poses serious risks concerning terminal decisions such as the approval or rejection of therapies. Cognitive distortions about statistics likely stem from poor teaching in schools and universities, overly simplified interpretations, and – as we suggest – the reckless use of calculation software with predefined standardized procedures. In light of this, we present a framework to recalibrate the role of frequentist-inferential statistics within clinical and epidemiological research. In particular, we stress that statistics is only a set of rules and numbers that make sense only when properly placed within a well-defined scientific context beforehand. Practical examples are discussed for educational purposes. Alongside this, we propose some tools to better evaluate statistical outcomes, such as multiple compatibility or surprisal intervals or tuples of various point hypotheses. Lastly, we emphasize that every conclusion must be informed by different kinds of scientific evidence (e.g., biochemical, clinical, statistical, etc.) and must be based on a careful examination of costs, risks, and benefits.

## Premises

“Why do so many colleges and grad schools teach α = 0.05?^1^ Because that's still what the scientific community and journal editors use. Why do so many people still use α = 0.05? Because that's what they were taught in college or grad school.” Wasserstein and Lazar [50], representatives of the American Statistical Association (ASA), chose to start their article on the ASA's official statement on the *P*-value with this declaration by Professor George Cobb (Professor Emeritus of Mathematics and Statistics at Mount Holyoke College). As widely reported in the literature [5,7,26,33,35,42,43,44], the *P*-value is a measure widely misused in public health. Specifically, such a number is mistakenly adopted to draw conclusions or make decisions concerning a real phenomenon (e.g., the effectiveness of a therapy) based on the usually arbitrary rule P < α = “statistical significance,” P ≥ α = “statistical non-significance” (when dealing with discrete data, the equality sign is included in the first inequality). Often, the term “significance” – an English synonym for “relevance” or “importance” – is used without the adjective “statistical,” thus leading many readers and scientists to confuse a mere mathematical outcome with scientific evidence [10]. Indeed, as noted by Karl Pearson [39], the absence of “statistical significance” does not imply the absence of association. McShane and Gal [34], Gelman [15], and Rafi and Greenland [41] highlight various possible biases and cognitive distortions that could drive the unhealthy success of “statistical significance,” including conflicts of interest and oversimplifications. However, also according to our personal experiences in learning and teaching, we note a possible overlooked aspect: the proliferation of software that, by removing all calculation procedures from scientists' perception, promotes an interpretation of results disconnected from the statistical, scientific, and epistemic requirements needed to attribute meaning to them [9,47]. To address these long-standing problems, the present work discusses the foundation of frequentist-inferential statistics as a means to help inform scientific conclusions and decisions.

## General foundation and scope of statistics

Hennig [27] describes various domains of reality that should be considered in properly defining science. Firstly, it is essential to recognize that our experience and even knowledge of any observer-independent reality passes through personal perception (incommunicable to other individuals in its exact state) and – as we would like to add – information mediators (e.g., photons, academic journals, mass media, etc.). In this context, science can be understood as a social system aimed at the realization of a growing body of stable constructs on which a certain degree of general agreement can be reached. As outlined by Good [52], every scientific theory must be based on a series of axioms, rules of application, and technical suggestions. For example, the functioning of the scientific method depends entirely on the likelihood of the principle of uniformity, according to which the laws of the universe are sufficiently stable to allow for equivalent replications (an aspect not taken for granted in a constantly changing world, see [45,46]. The theory of probability, in particular, playing an intermediate role between logical and empirical sciences, is a discipline deeply influenced by human psychology both in its application and formulation. Although so-called degrees of belief are primarily associated with the Bayesian framework, even frequentist statistics are built on the concept of expectation [26]. For instance, when flipping a fair two-sided coin, the idea of getting heads 50% of the time, in the long run, derives from a logical expectation: the probability (parameter) “ratio between favorable (1) and possible cases (2).” The trust we put in such a belief is so large that we usually test the fairness of a coin based on the latter, i.e., we are supposed to observe such a 50% frequency in a large number of equivalent repetitions (population) if and only if the coin is unbiased. Furthermore, it is often the case that we do not know the parameter “probability of an event” in the population of interest (e.g., the rate and degree of effectiveness of an antihypertensive drug in a population of hypertensive individuals). Since it is often impossible to analyze such a population, both for practical and ethical reasons (e.g., lack of resources to adequately follow up), it is possible to extract a sample from it. The methods used to carry out this procedure must aim to ensure its representativeness, i.e., that the variety of clinical and epidemiological situations contained in the population is accurately reflected in the selected sample. This is one of the purposes of the so-called inferential statistics, which requires numerous assumptions to work properly [31].

## Statistical models are even less than equations and Greek letters

Greenland et al. [26] loudly stated that a statistical model is more than equations and Greek letters, emphasizing the indispensable need to consider a broad set of scientific and epistemological assumptions to make it valid. We perfectly agree with this point. However, from an educational perspective, based on our experience with common misunderstandings in this regard, we think that a statistical model is even less than what could be believed. Specifically, a statistical model is just a set of rules pertaining to numbers. Even when we apply a descriptive approach, assigning numbers to observations (e.g., LDL cholesterol values) and calculating others based on the former (e.g., measures of central tendency and dispersion), we attempt to find a simple mathematical expression of a complex scientific phenomenon. In other words, the so-called raw data represent a set of symbols (numbers) devoid of intrinsic meaning (it is the researcher who knows their plausible relevance within a well-specified context). These mere symbols eliminate all perceptual aspects that might be part of a natural human intuition for better (e.g., sensory evaluation of symptoms and the overall health status of a patient) and for worse (e.g., biases and harmful beliefs about the patient or their condition). But not only that: those numbers are also influenced by subjectivity and psychological mechanisms (which come into play in all decisions regarding how to design the study, set up the experiment and measurement tools, etc.), which are difficult – if not impossible – to model [19,22]. The biochemical and physical laws that regulate or interfere with such phenomena are also largely omitted. Moreover, descriptive statistics like mean values and variances entail further loss of information as they compress a large multitude of numbers into very few. The goal is clearly to simplify an otherwise inextricable situation; however, it must be clear that such simplification is not without costs [43].

## It is all about context, risks, costs, and benefits

Regardless of their differing positions on various issues, the majority of statisticians and public health experts agree that every decision (or every method adopted to reach such decision) should be based on careful consideration of context, costs, risks, and benefits [3,15,17,24,26,29,32,36,37,49,52]. As stressed by the American Statistical Association, no statistical criterion can replace critical reasoning in this scope [50]. The importance commonly assigned to the decision-making role of critical reasoning appears to be proportional to the perception of the severity of the consequences of an incorrect decision (especially if this involves the authors directly; see [34]). For instance, the heuristics – generally taken as scientific standards – of trying to limit the number of false positives (Type I errors) to 5% and the number of false negatives (Type II errors) to 10% or even 20% collapse when faced with the real case of a metal detector (imagine the consequences of allowing 20% of armed individuals to pass through freely). Furthermore, it must also be clear that statistical assistance is often not necessary when the causal mechanisms underlying a process are well-known or even obvious (e.g., there is no urgent need for randomized clinical trials for parachutes; see [51]) and that scientific generalization is broader than mathematical description [10]. Thus, statistics is properly used only if employed to help inform – and not make – a decision. Conversely, the decision-making process must necessarily undergo what is called “decision analysis” [28,29].

## Fundamentals of the frequentist-inferential statistics

A statistical model enriched with interpretative character – implying a certain degree of subjectivity [16,19] – can be defined as a set of hypotheses fixed as true a priori [26]. For this reason, these are often referred to as model assumptions. Such assumptions are tasked with adequately representing, mathematically, a series of scientific and epistemological validity requirements [43,45]. Some are simply supposed true, as there are no well-defined investigative methods at the statistical level (Table 1). Others, however, can be examined and approached through a well-outlined set of procedures. The relationships between the various assumptions in the statistical plane and those in the empirical plane are mediated by the researcher's overall ability to model the scientific-epistemological request of validity.

**Table 1:** Relationship between statistical and scientific hypotheses.

Statistical hypotheses	Scientific and epistemic hypotheses
Not assessable (supposed true)	Principle of uniformity
Not assessable (supposed true)	Absence of human bias
Not assessable (supposed true)	Data integrity and appropriateness for the scope
Not assessable (supposed true)	Absence of unknown scientific uncertainties
…	…
Random sampling and sample size determination	Sample representativeness
Data normality and absence of outliers (generally required in parametric tests)	Absence of interpretative confounders
…	…
Target hypothesis (e.g., null hypothesis)	Scientific hypothesis (e.g., no pharmacological effect)

In this regard, a first evident and ineliminable factor of uncertainty is indeed inherent in such an overall ability. The assumption of primary interest is the so-called “target hypothesis,” which aims to incorporate into the statistical model the prediction of the scientific hypothesis regarding the phenomenon under investigation (e.g., the ineffectiveness of a therapy). However, the definition of this hypothesis is subject to serious controversies and criticisms within the public health realm.

## A practical example to understand the divergence P-value

**Fig. 1:**
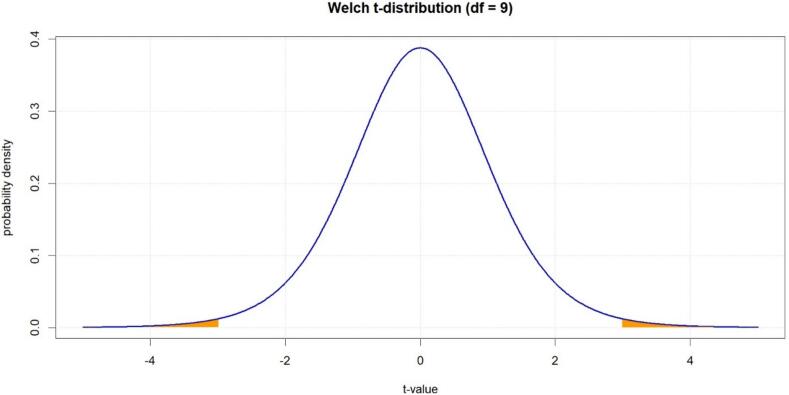
Welch t-distribution example (*df* = 9, t* = −2.96). The orange area corresponds to *P* = 0.016.

Let's suppose we are operating in a utopian scenario where all the so-called “background assumptions” (i.e., all hypotheses of the model excluding the target hypothesis) are perfectly true [43], and let's consider the example of a one-sample two-sided *t*-test. In particular, let's suppose we have measured an average blood pressure change after administering a certain therapy in a group of *n* = 10 hypertensive patients to be $\bar{x}$ = −15 mmHg (with a sample standard deviation, or *s*, of 16). The test statistic for the chosen distribution is calculated using the following formula:

$$t^{*}=\left(\bar{x}–\mu \right)/\left(s/\surd n\right)$$

where *μ* is the expected average variation. Under the mathematically null hypothesis of zero effect (which should represent the “no efficacy” hypothesis on the statistical plane), *μ* = 0. The expression “mathematically null” emphasizes that, in this case, the test assumes *μ* to be exactly 0 (e.g., *μ* = 0.001 mmHg is a mathematically non-null hypothesis despite the practically null effect it represents).^2^ It is reiterated that such a target/null hypothesis is also assumed to be perfectly true before calculating any result. From now on, we also refer to the calculated t* value with the expression “experimental statistical result” since it depends on the data measured or calculated in the experiment. The key point is that we have further condensed our experimental data into the single number t*. The distribution we have adopted doesn't consider in any way what happened in the experiment but only two statistics: the t* value we have generated (which has no memory of how it was generated) and the calculated degrees-of-freedom value *df* (which, in this specific test, merely depends on sample numerosity, i.e., *df* = *n –* 1). In other words, the whole statistical model – solely made of rules and numbers – has no information about the real phenomena involved in our experiment, and its epistemic validity strictly depends on our overall assessment capacity. Moreover, the statistical model assumes that chance is the only phenomenon at play. Thus, by substituting our experimental data and the mathematically null hypothesis in the above equation, we get t* = (−15–0)/(16/√10) = −2.96 and *df* = 10–1 = 9. After that, by calculating the area under the identified distribution outside the bounds defined by -t* and t*, i.e., *P* = Pr(|t| > |t*|) (Fig. 1), we obtain the so-called “divergence *P*-value” [23,30]. In our case, this is *P* = 0.016 (0.008 under the left tail plus 0.008 under the right tail).

The divergence P-value is the probability of obtaining, in infinite equivalent utopian repetitions (in which chance is the only factor at play), a test statistic as or more extreme than the one obtained in our experiment. In this sense, the divergence P-value can be interpreted as a continuous measure, conditional on the background assumptions, of the statistical evidence against the mathematical target hypothesis as evaluated by the chosen test. The term “divergence,” in line with the ASA statement, explicates that this *P*-value can be adopted as an index of the discrepancy of the experimental data from the prediction of the statistical model through the chosen statistical test. The closer the P-value is to 0, the higher the discrepancy; the closer it is to 1, the lower the discrepancy. For this reason, the clearest way to interpret the *P*-value is as a continuous measure of compatibility between the model prediction and the data. The closer it is to 0, the lower the compatibility; the closer it is to 1, the higher the compatibility [4,22,23,26,33]. In our example, *P =* 0.016 indicates that – as evaluated by the adopted test and conditionally on the background assumptions (which we assume to have well-validated) – our data have a low degree of compatibility with the chosen hypothesis (in this case, the mathematically null hypothesis *μ =* 0).

## An overview of the major criticalities

### Statistical criticalities

In a concrete application, the relationship between the data and the target hypothesis risks being obscured by violations of the background assumptions. Referring back to Table 1, here's how it works: generally, according to a certain statistical model, the P-value measures the conditional compatibility of the target hypothesis prediction (the supposed test statistic) with the experimental statistical result (the calculated test statistic). In simpler terms, the target hypothesis generates a certain prediction (e.g., *t = 0* under the mathematically null hypothesis), and the P-value measures the conditional compatibility of that prediction with the experimental statistical result (e.g., t*). If we want this evaluation to be solely related to the statistical target hypothesis, we must ensure that all other hypotheses (e.g., data normality, random sampling, absence of outliers, etc.) are at least valid! If this does not occur, these underlying hypotheses will directly intervene in such an evaluation by acting as potentially highly impacting confounding factors. In this regard, in addition to standard procedures for examining the validity of such assumptions, bias and sensitivity analysis approaches can be adopted to analyze the susceptibility of data and results to possible violations [21,29].

### Epistemological criticalities

It is essential to keep in mind that frequentist-inferential statistics operate in an idealized, mathematically perfect world made of pure chance (better understood as “total absence of causal mechanisms”). Therefore, the choice of the statistical model (i.e., the choice of all underlying hypotheses and associated procedures) is crucial to reach a properly informed scientific conclusion. In other words, the selected approach must be the best option for introducing the scientific hypotheses into the mathematical framework. For example, random sampling is the statistical technique used to attempt to obtain a truly random sample (statistical plane), which in turn is used to attempt to obtain a sample that is sufficiently representative of the real population (scientific-epistemic plane). In an epidemiological study, such a sample should encompass all possible contextual peculiarities (e.g., clinical conditions that are concomitant or correlated to the investigated phenomenon, exposure to or presence of risk factors, etc.) because the aim is to generalize the result to the entire population (which, of course, is composed of a large amount of “particular cases” called “people”). The goal is to maintain in the sample the same relative frequencies (of properties of interest) that characterize the population. The same applies to randomization (a procedure to achieve random sampling). Imagine a salad made of 1/3 lettuce leaves, 1/3 tomatoes, and 1/3 corn. If I layer these ingredients in that order from the bottom up and scoop superficially, I'll only get corn (which doesn't represent my “salad” population well). But, if I mix them thoroughly first (randomization), then even a small scoop (ideally containing all frequencies of 1/3 of the previous ingredients) would be a good representation of the entire “salad” population. However, as stressed by Fisher [14] and reiterated by Rubin [45], in such a complex scientific context, it's highly implausible that the parameters of interest remain sufficiently constant over time or that a population can be uniquely defined (which is sufficient to prevent equivalent replications of experiments). The inability to know these (and other) uncertainties and sources of uncertainty makes statistical inference uncertain in turn and generally overconfident or even dangerous when based on individual studies [3,5,8,22,42,43].

### Scientific criticalities

Despite its vital importance, the choice of a target hypothesis capable of well describing a scientific question is one of the most trivialized or even ignored aspects in specifying a statistical model. The current fashion is to select by default the target hypothesis of no effect (although the target hypothesis “the treatment is effective” would seem the best choice in clinical settings). However, the most devastating issue is that the usual statistical transposition of such a scientific hypothesis is the so-called statistically “null hypothesis” of a perfectly zero effect. For example, the statistically null hypothesis in the context of a treatment applied to a test group A and a placebo group B might be as follows: the difference between the mean values of the clinical outcomes of A and B is exactly 0. This means that any extremely small difference on the clinical level but non-zero on the mathematical level (e.g., 0.1, 0.2, 0.3) is not considered by the model as the null hypothesis! Therefore, there are infinite possible situations in which practically irrelevant results give rise to extremely small *P*-values, leading authors to erroneously and unfoundedly conclude that an effect exists (or, for the more cautious but still mistaken, that there is evidence in favor of an effect). Reconnecting to the one-sample *t*-test to investigate the drop in blood pressure in hypertensive patients before and after treatment, if a mean reduction of 0.1 mmHg is measured with a standard deviation of 0.1 mmHg in a group of 10 patients under the null hypothesis of zero effect, *P =* 0.01 is obtained (i.e., the statistical result showed very low compatibility with the null hypothesis of a perfectly zero effect despite the clinically negligible effect). Besides, the efficacy of therapy encompasses much more complex aspects than a simple equation, such as economic sustainability, applicability, physical and psychological invasiveness, side effects, and the duration and extent of benefits [40].

## The solution is to openly describe what you observe and wait for other evidence

According to Amrhein, Trafimow, and Greenland [7], “There Is No Replication Crisis if We Don't Expect Replication.” Specifically, since experiment replications are generally variable (or, at least, equivalence cannot be guaranteed) and it is not possible to infer based on individual studies, the solution is to adopt a descriptive-unconditional approach [5,7] while waiting for multiple solid evidence [2]. The term “compatibility” (dating back to Karl Pearson [38]) requires a much less stringent condition than “support” since logically different hypotheses can be compatible with a result. For example, finding a person at the crime scene is compatible with their guilt as well as with an attempt to provide assistance. Alongside this, the unconditional approach implies a cognitive and ethical effort not to favor hypotheses of greater scientific interest over hypotheses of greater scientific relevance. This means that the scientist must strive to present all scenarios most compatible with the data (e.g., limitations) equivalently and not just those that are potentially more appealing (a problem sharing elements with detrimental dynamics such as publication bias and P-hacking, [43]). In light of the unfeasibility of guaranteeing perfect impartiality, let's correct this terminology by adding the word “quasi,” i.e., quasi-unconditional approach. In this context, inference is not necessarily abandoned but simply requires a high number of studies with compatible (coherent, consistent, concordant, consonant) results. The expectation is that the goal of various research groups to minimize the impact of the uncertainty sources may render the statistical errors subject to an acceptable degree of randomness, thus not invalidating the data in the long run (or, better, over numerous repetitions). Such an expectation is much more realistic than being able to infer from individual studies or to limit – as in the Neyman-Pearson approach – the number of false positives (Type I errors) and negatives (Type II errors) to specific thresholds (the presence of variability errors, sometimes noted as Type III errors, mathematically invalidates this possibility, see [6,7,45], and [22,23,48]). Moreover, since statistics alone does not have probative value, it is essential to compare statistical outcomes with evidence of other nature (e.g., biochemical, clinical, physical, psychological, etc.) and observe if all of these agree with each other [8,12,21,43].

## How to properly read statistical compatibility

Within the statistical compatibility approach, the conventional “confidence intervals” become “compatibility intervals” as they report the set of hypotheses more compatible with the data – as conditionally assessed by the chosen test – compared to certain threshold hypotheses (those occupying the boundaries of the interval). Let's now illustrate a series of key examples. From now on, we refer to the *P*-value for the mathematically null hypothesis of zero effect as the “null P-value” or “null *P*:” By doing so, it will be clear to the reader that this P-value concerns solely the hypothesis of a null effect.

Example 1

Suppose we have measured an experimental hazard ratio (HR*) equal to 3, with a 95% compatibility interval of 95% CI = (1, 9), null *P* = 0.05. Many would, in our view erroneously, conclude “statistical non-significance” for equivalently wrong reasons, such as “the null hypothesis HR = 1 is contained within the 95% CI” and “null *P* ≥ 0.05”.^3^ However, HR = 1 and HR = 9 are equally compatible with the data according to the statistical model since, as endpoints of a 95% CI, the associated P-value is 0.05 in both cases. Therefore, our data are equally consistent with hypotheses of a negligible effect (HR = 1) and a highly harmful effect (HR = 9). Additionally, the crucial point is that the hypothesis most compatible with the data given the assumptions is HR = HR* = 3 (*P* = 1). This means that, according to the selected test, the experimental HR* = 3 is perfectly compatible with the target hypothesis Ht: HR = 3. Thus, although subject to some statistical uncertainty, this scenario is more compatible with hypotheses of harmful effects rather than absent effects.

Example 2

Suppose we have measured an experimental HR* = 1.07, 95% CI = (1.01, 1.13). Many would, in our view erroneously, conclude “statistical significance” for equivalently wrong reasons, such as “the null hypothesis HR = 1 is not contained within the 95% CI” and “null *P* < 0.05.” However, this 95% CI shows that the hypotheses most compatible with our data – as evaluated by the statistical model – are those predicting effects that can be considered small in many contexts. Furthermore, the best point estimate itself (HR* = 1.07) aligns with a generally small effect size. Therefore, regardless of the *P*-value for the mathematically null hypothesis, these outcomes are more compatible with hypotheses of marginal effects. Nonetheless, it is essential to recognize that 95% compatibility intervals this narrow signal a conditional statistical precision but not scientific precision, understood as the extent to which a study produces results consistent with the scientific phenomenon under investigation. Specifically, scientific precision is one of the assumptions on which compatibility intervals are constructed. Moreover, since narrow intervals can also be obtained under violations of the underlying assumptions, they do not reflect the quality of the model validation.

Example 3

Suppose we have measured an experimental HR* = 1, 95% CI = (0.1, 10.0). Many would, in our view erroneously, conclude “statistical non-significance” for equivalently wrong reasons, such as “the best point estimate is HR* = 1” and “the null *P* = 1.” However, the width of the 95% compatibility interval is so wide that hypotheses of a protective effect (e.g., HR = 0.4 or HR = 0.5) and hypotheses of a harmful effect (e.g., HR = 2 or HR = 3) are also very compatible with the data as evaluated by the chosen statistical model. Consequently, although the most consonant result is that of a null effect, these outcomes align with a marked statistical uncertainty, thus affecting the formulation of a first conditional conclusion about the examined phenomenon. In other words, even assuming all procedures have been perfectly performed, statistical uncertainty necessarily implies scientific uncertainty due to the impossibility of determining which scientific hypothesis (e.g., small, medium, or large effect size) is most compatible with the data.

Example 4

Suppose we have measured an experimental HR* = 0.4, 95% CI = (0.2, 0.7). Since all hypotheses contained within the 95% CI and the best point estimate itself are consistent with a protective effect (on the mathematical level), we can conclude that—conditionally on the background assumptions—these results are compatible with a protective effect (regardless of the *P*-value for the mathematically null hypothesis). However, this is far from providing evidence of causality.

Example 5

**Fig. 2:**
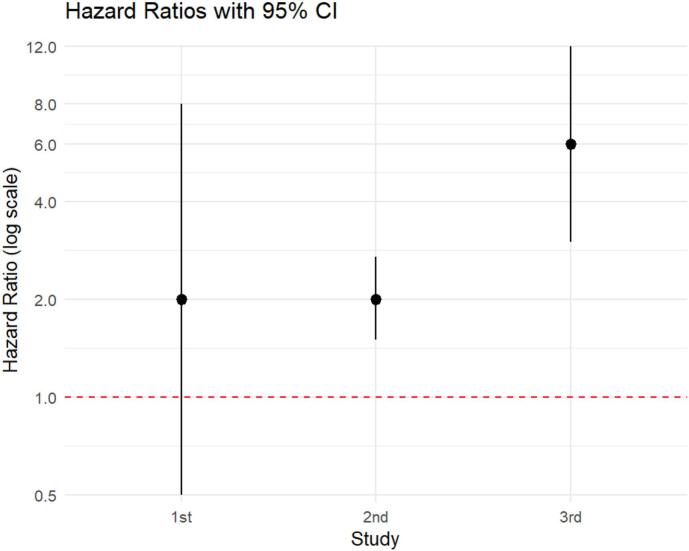
Hazard ratios and 95% compatibility intervals for three different studies (replications).

Suppose we have the following hazard ratios and their 95% compatibility intervals (Fig. 2). Let's also assume that these outcomes pertain to replications of the same study. Many would, in our view erroneously, conclude that the first study yielded a “statistically non-significant” outcome (since the null hypothesis belongs to the 95% CI) while the other two yielded a “statistically significant” outcome (since the null hypothesis does not belong to the 95% CI). In other words, according to the above-mistaken interpretation, the last two studies would be in agreement with each other and both in disagreement with the first one.

However, the first two studies are in perfect agreement with each other because the hypothesis most compatible with the data given the assumptions is HR = 2 in both cases (it's easy to find a test for which the P-value for the mathematically null hypothesis of no difference between the two studies is exactly 1). Conversely, the third study is quite inconsistent with the first two because the hypothesis most compatible with the data given the assumptions is HR = 6, i.e., three times that of the previous two studies. More specifically, the degree of disagreement is more moderate in comparison 1 vs. 3 due to the statistical uncertainty affecting both results (very wide 95% compatibility intervals), while the degree of disagreement is very strong in comparison 2 vs. 3 (as the 95% compatibility intervals do not overlap). Alongside this, it's important to note that these comparisons are conditional on the background assumptions of all three studies! Therefore, at first glance, the results appear to be more compatible with a harmful effect, although the size of such an effect is still very unclear (this calls for further investigations with greater control over sources of uncertainty). We note that the generally most appropriate way to proceed in these cases is to conduct a meta-analysis. For example, using a random effects model, this scenario produces the following results: HR* = 2.9, 95% CI = (1.3; 6.7), percentage of heterogeneity I^2^ = 76%, 95% CI = (20%, 93%). We thus see that the situation is consistent with the previous preliminary conclusion. Nonetheless, we point out that, when implementing and subsequently interpreting large meta-analyses, a careful examination of the specific situation and other characteristics (e.g., effect types and publication bias) is required. The following works may be useful for this purpose [1,13,18].

### Example 6: how to calculate compatibility intervals

**Fig. 3:**
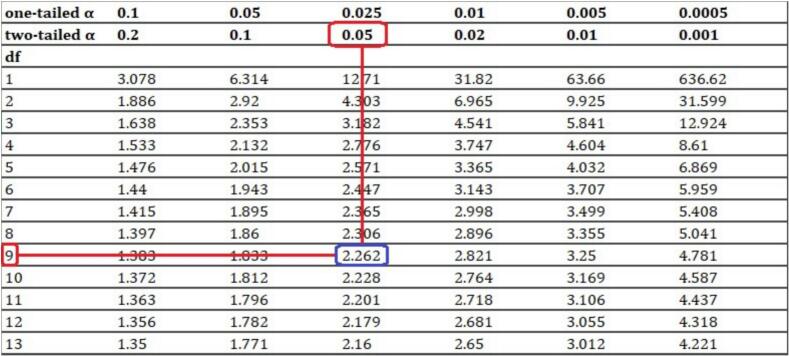
Critical values (*t*_*c*_) for the t-distribution.

Let's reconsider the *t*-test (e.g., Fig. 1) and suppose we want to calculate the associated (1 *–* α) · 100% compatibility interval (e.g., 95% CI). First, we need to find the positive critical value *t*_*c*_ for which the area under the distribution with *df* degrees of freedom between *-t*_*c*_ and *t*_*c*_ is equal to (1 *–* α) · 100% of the total unit area. To do this, we simply need to impose that the integral of such a distribution *f(x)* in *dx* between *-t*_*c*_ and *t*_*c*_ is equal to 1 *–* α (these critical values are generally tabulated as a function of degrees of freedom, see Fig. 3). Since *t*_*c*_ can be expressed as *t*_*c*_ = |*x̅ – μ*|/(*s*/*√n*), we have |*x̅ – μ*| = *t*_*c*_ *s*/*√n*. It follows that *μ*_*1,2*_ = *x̅ ± t*_*c*_ *s*/*√n*. In other words, given the symmetry of the distribution, there are always two target hypotheses (*μ*_*1,2*_) that yield the same critical t-value (*t*_*c*_). In order to obtain the set of target hypotheses *μ* that yield *t* < *t*_*c*_ (thus, hypotheses more compatible with the data than the hypotheses *μ*_*1,2*_), we need to solve the inequality |*x̅ – μ*|/(*s*/*√n*) < *t*_*c*_, which leads to the interval (*μ*_*1*_, *μ*_*2*_) = (*x̅ – t*_*c*_ · *s*/*√n*, *x̅ + t*_*c*_ · *s*/*√n*). So, if we want to calculate a (1–0.05) · 100% = 95% compatibility interval for the example associated with Fig. 1 (*df* = 9), we have *t*_*c*_ = *t*_*0.95*_ = 2.262 (see Fig. 3). After that, we find 95% CI = (*x̅ – t*_*0.95*_ · *s*/*√n*, *x̅ + t*_*0.95*_ · *s*/*√n*) = (−15–2.262 · 16/*√*10, −15 + 2.262 · 16/*√*10) = (−26, −3.5). The same operation can be done to calculate, for instance, a (1–0.02) · 100% = 98% CI (*t*_0.98_ = 2.821, see Fig. 3) or any other desired (1 *–* α) · 100% compatibility interval.

## How to properly report statistical compatibility

The preferred choice of a 95% compatibility interval over other intervals (e.g., 97%, 91%, 85%, 75%, etc.) lacks a statistical or scientific foundation but is the result of a misinterpreted suggestion by Sir Ronald Fisher (the famous 5% “statistical significance” threshold). A compatibility interval of (1-α)100% aims to display all hypotheses with a *P*-value > α, i.e., all hypotheses more compatible with the data than the threshold hypotheses (the interval boundaries, i.e., those with P = α) as conditionally evaluated by the adopted statistical test [26,33,[41], [42], [43]]. Consequently, under the same premises as mentioned above, hypotheses outside the interval are less compatible with the data compared to those inside the interval. On this point, it should be noted that all values inside are not equally compatible; the point estimate is the most compatible, and values near it are more compatible than those near the interval limits. Similarly, not all values outside are equally (in)compatible. The values just outside the limits are practically just as compatible as those just inside the limits but those far from the limits are much less compatible. Consequently, although it provides more information than the *P*-value for the mathematically null hypothesis, a single compatibility interval offers a limited perspective of the statistical compatibility picture. Indeed, we lack direct, clear information about the exact relationship between the data and the hypotheses within the interval (except for the hypothesis equivalent to the best point estimate, whose *P* = 1). To mitigate this issue, Amrhein and Greenland [3] suggest plotting compatibility curves and observing how the P-value changes over a wide continuous range of hypotheses. This solution provides a complete scenario but also has some drawbacks. One of these is certainly the impossibility of being included in an abstract or other forms of concise communication of results. On this point, Rovetta [42,43] suggests adopting a new convention for reporting multiple intervals, thus finding a compromise between the demand for more information and the need for greater communicability. For instance, concerning the one-sample *t*-test for the hypothetical hypertension treatment (Fig. 1), three compatibility intervals of the form 98% CI = (−29, −1), 95% CI = (−26, −4), 90% CI = (−24, −6) can be represented as follows: 98|95|90% CI = (−29, −1| -26, −4| -24, −6). By doing so, it is possible to observe various degrees of conditional compatibility depending on various hypotheses of mathematical effect size. For example, we know that hypotheses *μ* = −29 mmHg (large effect) and *μ* = −1 mmHg (clinically irrelevant effect) are poorly compatible with the data (*P* = 0.01), while hypotheses *μ* = −26 mmHg (large effect) and *μ* = −6 mmHg (small effect) are more compatible (*P* = 0.10). In light of the best point estimate *x̅* = −15 mmHg, these findings are consistent with the existence of some efficacy as well as with the presence of confounding factors (largely due to the absence of a placebo group) or, since the study is single, with a mere statistical coincidence. Indeed, the whole context should also be sustained by solid evidence of other nature, such as a recognized scientific principle (e.g., a known biochemical mechanism) to support causality. And again, any terminal decision should be based on a thorough evaluation of costs, risks, and benefits. Another possible way to present the results is to show the compatibility (*P*-value) associated with various hypotheses of interest, which must be selected a priori before conducting the experiment (protocol, to be published with a DOI). In this regard, we can adopt the notation *μ*_*1*_|*μ*_*2*_ = *P*_*1*_|*P*_*2*_. For instance, setting the hypotheses *μ*_*1*_ = 0 (null effect), *μ*_*2*_ = −5 (small effect), *μ*_*3*_ = −10 (medium effect), *μ*_*4*_ = −15 (satisfactory effect), *μ*_*5*_ = −20 (optimal effect), we have: 0|-5|-10|-15|-20 = 0.02|0.08|0.35| 1 |0.35: The hypotheses most compatible with the data are those that predict a satisfactory effect (according to the conditional evaluation of the test).

## How to properly quantify statistical information

As extensively argued in the previous paragraphs, assuming all underlying hypotheses are true, *P*-values are continuous measures of the data compatibility with the target hypothesis as assessed by the chosen test: P-values close to 1 (respectively 0) indicate high (respectively low) compatibility. At the same time, P-values can equivalently be used as indices of incompatibility: P-values close to 0 (respectively 1) indicate high (respectively low) incompatibility. Again, the term incompatibility can also be interpreted as “unexpectedness” or “surprise:” A result that is highly incompatible with a model prediction is indeed a very surprising result. However, these definitions risk being too vague as they do not provide clear indications for quantitative evaluations. For example, how much more surprising is the hypothesis associated with *P*_*1*_ = 0.02 compared to the hypothesis associated with *P*_*2*_ = 0.04? The answer is quite simple if we imagine the *P*-value as a probability: in an ideal world where no scientific phenomenon exists beyond mere chance, the first result is as surprising as an event that happens 2 times out of 100 (*P*_*1*_ = 0.02, i.e., 2%), while the second result is as surprising as an event that happens 4 times out of 100 (*P*_*2*_ = 0.04, i.e., 4%). Thus, we are dealing with probabilities where one is twice the other, i.e., *P*_*2*_*/P*_*1*_ = 2. Nonetheless, this interpretation also risks eluding our cognitive abilities due to the difficulty of relating everyday events – of which we have immediate perception – with frequencies or, even worse, frequency ratios. Such a risk is further compounded by the fact that information in a probability, including a P-value, is log-inverse scaled. To solve these problems, it is possible to equate P-values to the probability of obtaining “S” consecutive heads when flipping a fair two-sided coin: *P* = 0.5^*S*^, from which it follows *S* = log_0.5_ (*P*) = −log_2_ *P*. This value of *S*, or S-value, is called surprisal [3,11,20,25,33,[41], [42], [43]]. By doing so, we observe that *S*_*1*_ = −log_2_ (0.02) = 5.6 (almost 6 consecutive heads) and *S*_*2*_ = −log_2_ (0.04) = 4.6 (almost 5 consecutive heads). Therefore, the difference in surprise between these two outcomes is equivalent to the surprise we experience in obtaining one head when flipping a coin (since 5.6–4.6 = 1). Since the S-value helps us quantify statistical information, we will use the “bit” as the unit of measurement (e.g., 3 bits = 3 consecutive heads). Based on this framework, we propose two possible approaches: one very recent and innovative and the other less innovative but still useful!

### A recent, innovative approach

This involves replacing *P*-values and compatibility intervals with S-values and surprisal intervals (or S-intervals) respectively, where the latter represents the set of hypotheses less surprising than “S” consecutive heads (flipping a fair coin, according to the adopted test). For example, suppose we want to present a statistical summary for a Pearson correlation with an experimental point estimate of R* = 0.92 and a null P-value of 0.01 (*n* = 6). Using the formula *S* = −log_2_ (0.01) = 6.6 bits. Now, let's calculate a 3-interval (3-I), which is the set of hypotheses less surprising than 3 consecutive heads compared to R* = 0.92. This involves calculating an appropriate compatibility interval (1-α)100% CI: indeed, α is a specific P-value threshold we have established, which can also be interpreted as the probability of obtaining S_α_ consecutive heads using the formula α = (0.5)^*S*α^. Given *S*_α_ = 3, we have α = (0.5)^3^ = 1/8 = 0.125. Hence, our 3-I = (1–0.125)100% CI = 87.5% CI, i.e., our 3-interval corresponds to the 87.5% compatibility interval! Therefore, we have 3-I = (0.59, 0.98), which indicates that the hypotheses *R* = 0.60, *R* = 0.61, or *R* = 0.97, *R* = 0.96, etc., being within the 3-I, are less surprising 3 consecutive heads compared to the experimental numerical result R* = 0.92 (as assessed by the test). Using the above convention, we can also evaluate multiple S-intervals such as 2|3|4-I = (0.72, 0.97|0.59, 0.98|0.46, 0.99) to observe how the surprise changes in relation to different parameter hypotheses [42,43]. In this regard, we particularly praise the work of Rafi and Greenland [41] on information graphs. As a side note, we want to further emphasize the practical equivalence of cases such as *P*_*1*_ = 0.04 (*P* < 0.05) and *P*_*2*_ = 0.06 (*P* ≥ 0.05), since *S*_*1*_ = −log_2_(0.04) = 4.64 bits and *S*_*2*_ = −log_2_(0.06) = 4.06 bits such that *S*_*1*_ – *S*_*2*_ = 0.58 bits (much less surprising than getting heads when flipping a coin). Finally, we point out to the reader that, thanks to the properties of logarithms, this comparison can also be performed as follows: *S*_*1*_ – *S*_*2*_ = log_2_ (*P*_*2*_*/P*_*1*_) [42,43]. A recent online calculator has been developed to swiftly calculate S-values and surprisal intervals.^4^

### A less innovative but still useful approach

Thanks to the relationship that compares *P*-values to obtaining *S* consecutive successes when flipping a fair coin, i.e., *P* = (1/2)^*S*^, it is easy to evaluate the information difference between two P-values through their ratio. Indeed, *P*_*2*_*/P*_*1*_ = (1/2)^*S2–S1*^ = 2^*S1–S2*^ = 2^|*∆S*|^. Conveniently placing the larger P-value in the numerator, for *P*_*2*_*/P*_*1*_ = 1, 2, 4, 8, 16, 32 we get |*ΔS*| = 0, 1, 2, 3, 4, 5, respectively. In other words, we just need to think of the powers of 2 that include the P-ratio to understand the difference in surprise between *P*_*2*_ and *P*_*1*_ (Table 2).

**Table 2:** Relationships between P-value ratios and information absolute difference (IAD). As a useful convention, it is assumed that the larger P-value is in the numerator of the ratio.

P-value ratio	Information absolute difference (bits)
From 1 to 2	From 0 to 1
From 2 to 4	From 1 to 2
From 4 to 8	From 2 to 3
From 8 to 16	From 3 to 4
From 16 to 32	From 4 to 5
From 32 to 64	From 5 to 6
From 64 to 128	From 6 to 7

Let's look at some practical examples. We evaluate the difference in surprise between the following pairs of P-values: P_1_ = 0.04, P_2_ = 0.06, P_3_ = 0.16. We have P_2_/P_1_ = 1.5, which is a number between 1 and 2, where 1 = 2^0^ and 2 = 2^1^. Therefore, we know that the difference in surprise between P_2_ and P_1_ is between 0 and 1 bits! P_3_/P_1_ = 4. Since 2^2^ = 4, we know that the difference in surprise between P_3_ and P_1_ is exactly 2 bits. And again, P_3_/P_2_ = 2.7, a number between 2 and 4, where 2 = 2^1^ and 4 = 2^2^. Hence, the difference in surprise between P_3_ and P_2_ is between 1 and 2 bits. Observing another ratio P″/P′ = 12.5, we note that 12.5 is between 8 and 16, that is 2^3^ and 2^4^, respectively; thus, the difference in surprise between P″ and P′ is between 3 and 4 bits.

### How to read information differences

Difference of S-values or *P*-value ratios with IAD serve to assess the “surprise” discrepancy between two or more statistical outcomes but do not provide a complete information summary. For example, the pairs {*P*_*1*_ = 0.001, *P*_*2*_ = 0.004} and {*P*_*3*_ = 0.20, *P*_*4*_ = 0.80} contain *P*-values that have the same “distance” in terms of bits (since *P*_*2*_*/P*_*1*_ = *P*_*4*_*/P*_*3*_ = 4, i.e., 2 bits), even though the first pair is made of very small P-values (they both signal high incompatibility, i.e., low compatibility) and the second pair is made of very large *P*-values (they both signal low incompatibility, i.e., high compatibility). And what does it mean that *P*_*2*_*/P*_*1*_ = 0.004/0.001 is 2 bits apart? It means that *P*_*2*_ provides 2 bits less information against hypothesis 2 than *P*_*1*_ provides against hypothesis 1. Therefore, information differences are an additional tool to quantify the relative (in)compatibility but are blind on the global information location. For this reason, it is always essential to report P-values (or S-values) in full.

## How to properly assess statistical compatibility to reach a communicable conclusion

There is neither a universally definable nor plausible scale for evaluating statistical compatibility. Muff et al. [36] proposed a qualitative assessment system that, if appropriately translated in terms of compatibility, in line with Amrhein and Greenland [4], can bring “more good than harm.” However, here, we propose an alternative to try to better explicate the relationship with costs, risks, and benefits (which must be appropriately evaluated starting from the study protocol design phase). In conclusion, we suggest executing the following steps:

i) Set a scientific context and clearly explain the research objective and the sought or involved causal processes (including an analysis of the previous literature on the subject). Carefully examine possible sources of uncertainty and bias. Establish a research protocol (where appropriate, specify effect size ranges based on costs, risks, and benefits). All this should be published before starting the study (even as a preprint with DOI).
ii) Validate, using all deemed appropriate methodologies, the background assumptions. Report the results of this assessment in a supplementary file to allow independent reading by readers. It is important to keep in mind that the statistical model takes into account many hypotheses (e.g., background assumptions) and does not give priority to the target hypothesis. The validation phase serves precisely to ensure that the test primarily assesses the statistical relationship between the data and the target hypothesis (rather than between the data and all hypotheses of the model).
iii) First and foremost, evaluate the best point estimate obtained (in a two-tailed test, the P-value for the corresponding hypothesis is always equal to 1). Additionally, the conditional compatibility of the data with other hypotheses of interest (e.g., null, low, medium, and strong clinical effects) should be assessed. Multiple compatibility intervals (or surprisal intervals) or tuples of hypotheses and P-values (or S-values) can be used to do this, as shown in the preceding paragraph. One-tailed tests are advisable if there are valid scientific reasons (e.g., biochemical mechanisms or a certain number of previous concordant experiments) to expect an effect in a specific direction.
iv) Keep in mind that *P* = 1 indicates the hypothesis most compatible with the data (as evaluated by the chosen statistical test, conditionally on the validity of its background assumptions). To assess the relative (in)compatibility between various hypotheses within the same statistical model (e.g., *P*_*1*_ = 0.10 and *P*_*2*_ = 0.30), use S-values (e.g., *S*_*1*_ *– S*_*2*_ = log_*2*_ *(P*_*2*_*/P*_*1*_*)* = 1.6 bits) or P-value ratios and the information difference scale (e.g., *P*_*1*_/*P*_*2*_ = 0.30/0.10 = 3, i.e., from 1 to 2 bits).
v) Equally present all scenarios consistent with the data, including limitations. This involves a proper mention in the abstract and conclusions. Indeed, although they could be less interesting than the target hypothesis, non-target hypotheses that are equally consistent with the outlined scenario carry the same scientific weight as the target hypothesis.
vi) Remember that statistics is a set of rules and numbers mathematically devoid of probative capacity. It is up to the scientist to interpret these tools appropriately, i.e., in relation to the scientific context and the practical costs, risks, and benefits.
vii) Do not make or suggest terminal decisions (e.g., “the treatment should not be approved” or “this evidence supports the approval of the drug”) based on single studies. Moderate conclusions (e.g., “based on our evaluations, these outcomes justify further research”) are always acceptable if they come from an honest, transparent, and competent analysis to the best of your abilities.

## CRediT authorship contribution statement

**Mohammad Ali Mansournia:** Visualization, Validation, Supervision, Resources, Methodology, Formal analysis, Data curation. **Alessandro Vitale:** Writing – review & editing, Writing – original draft, Visualization, Validation, Supervision, Resources, Project administration, Methodology, Investigation, Formal analysis, Data curation, Conceptualization.

## Declaration of competing interest

The authors declare that they have no known competing financial interests or personal relationships that could have appeared to influence the work reported in this paper.

The author Mohammad Ali Mansournia is an Editorial Board Member/Editor-in-Chief/Associate Editor/Guest Editor for *Global Epidemiology* and was not involved in the editorial review or the decision to publish this article. No other authors hold senior positions that could be perceived as influencing a decision about this manuscript.

## Footnotes

1 The original quote included the letter “p” instead of the letter “α.” However, since the comment is addressed at the threshold of “statistical significance,” we preferred to use the conventional notation.

2 In the context of ratios (e.g., the hazard ratio “HR”), the mathematically null hypothesis is “ratio = 1” (e.g., HR = 1). Every ratio that is not exactly 1 (e.g., HR = 1.001 or HR = 0.999) is a mathematically non-null hypothesis.

3 It is noted to the reader that we deem the expression “statistical significance” appropriate only when referring to the late Fisherian “strength of the evidence against the null hypothesis” in relation to a statistical test. On the contrary, the “significance level” of Neyman and Pearson pertains to a decision rule that acquires meaning only through numerous equivalent replications and does not concern the statistical relevance of a single outcome.

4 S-value online calculator: https://www.statskingdom.com/surprisal-calculator.html

## References

[1] Ackerman D.L., Greenland S. (2002). Multivariate meta-analysis of controlled drug studies for obsessive-compulsive disorder. J Clin Psychopharmacol.

[2] Amaral O.B., Neves K. (2021). Reproducibility: expect less of the scientific paper. Nature.

[3] Amrhein V., Greenland S. (2022). Discuss practical importance of results based on interval estimates and p-value functions, not only on point estimates and null p-values. J Inf Technol.

[4] Amrhein V., Greenland S. (2022). Rewriting results in the language of compatibility. Trends Ecol Evol.

[5] Amrhein V., Greenland S., McShane B. (2019). Scientists rise up against statistical significance. Nature.

[6] Amrhein V., Korner-Nievergelt F., Roth T. (2017). The earth is flat (p > 0.05): significance thresholds and the crisis of unreplicable research. PeerJ.

[7] Amrhein V., Trafimow D., Greenland S. (2019). Inferential statistics as descriptive statistics: there is no replication crisis if we Don’t expect replication. Am Stat.

[8] Bann D., Courtin E., Davies N.M., Wright L. (2024). Dialling back ‘impact’ claims: researchers should not be compelled to make policy claims based on single studies. Int J Epidemiol.

[9] Bolker B., Fox J. (2014, June 4). Guest post: is statistical software harmful?. Dyn Ecol.

[10] Boring E.G. (1919). Mathematical vs. scientific significance. Psychol Bull.

[11] Cole S.R., Edwards J.K., Greenland S. (2021). Surprise!. Am J Epidemiol.

[12] Dobler C.C., Guyatt G.H., Wang Z., Murad M.H. (2021). Users’ guide to medical decision analysis. Mayo Clin Proc.

[13] Doosti-Irani A., Nazemipour M., Mansournia M.A. (2020). What are network meta-analyses (NMAs)? A primer with four tips for clinicians who read NMAs and who perform them (methods matter series). Br J Sports Med.

[14] Fisher R. (1955). Statistical methods and scientific induction. J R Stat Soc Ser B Methodol.

[15] Gelman A. (2018). The failure of null hypothesis significance testing when studying incremental changes, and what to do about it. Personal Soc Psychol Bull.

[16] Gelman A., Hennig C. (2017). Beyond subjective and objective in statistics. J Roy Stat Soc Ser A.

[17] Gelman A., Stern H. (2006). The difference between “significant” and “not significant” is not itself statistically significant. Am Stat.

[18] Greenland S. (1994). Invited commentary: a critical look at some popular meta-analytic methods. Am J Epidemiol.

[19] Greenland S. (2012). Transparency and disclosure, neutrality and balance: shared values or just shared words?. J Epidemiol Community Health.

[20] Greenland S. (2019). Valid P-values behave exactly as they should: some misleading criticisms of P-values and their resolution with S-values. Am Stat.

[21] Greenland S. (2021). Analysis goals, error-cost sensitivity, and analysis hacking: essential considerations in hypothesis testing and multiple comparisons. Paediatr Perinat Epidemiol.

[22] Greenland S. (2023). Connecting simple and precise P-values to complex and ambiguous realities (includes rejoinder to comments on “divergence vs. decision P-values”). Scand J Stat.

[23] Greenland S. (2023). Divergence versus decision P-values: a distinction worth making in theory and keeping in practice: or, how divergence P-values measure evidence even when decision P-values do not. Scand J Stat.

[24] Greenland S., Hofman A. (2019). Multiple comparisons controversies are about context and costs, not frequentism versus Bayesianism. Eur J Epidemiol.

[25] Greenland S., Mansournia M.A., Joffe M. (2022). To curb research misreporting, replace significance and confidence by compatibility: a preventive medicine Golden Jubilee article. Prev Med.

[26] Greenland S., Senn S.J., Rothman K.J., Carlin J.B., Poole C., Goodman S.N. (2016). Statistical tests, P values, confidence intervals, and power: a guide to misinterpretations. Eur J Epidemiol.

[27] Hennig C. (2010). Mathematical models and reality: a constructivist perspective. Found Sci.

[28] Kent D.L. (1992). The basics of decision analysis. J Dent Educ.

[29] Lash T.L., Fox M.P., MacLehose R.F., Maldonado G., McCandless L.C., Greenland S. (2014). Good practices for quantitative bias analysis. Int J Epidemiol.

[30] Lehmann E.L., Lehmann E.L. (2011). Fisher, Neyman, and the creation of classical statistics.

[31] Mansournia M.A., Collins G.S., Nielsen R.O., Nazemipour M., Jewell N.P., Altman D.G. (2021). A CHecklist for statistical assessment of medical papers (the CHAMP statement): explanation and elaboration. Br J Sports Med.

[32] Mansournia M.A., Nazemipour M. (2024). Recommendations for accurate reporting in medical research statistics. Lancet (London, England).

[33] Mansournia M.A., Nazemipour M., Etminan M. (2022). P-value, compatibility, and S-value. Glob Epidemiol.

[34] McShane B.B., Gal D. (2016). Blinding us to the obvious? The effect of statistical training on the evaluation of evidence. Manag Sci.

[35] McShane B.B., Gal D. (2017). Statistical significance and the dichotomization of evidence. J Am Stat Assoc.

[36] Muff S., Nilsen E.B., O’Hara R.B., Nater C.R. (2022). Rewriting results sections in the language of evidence. Trends Ecol Evol.

[37] Neyman J. (1977). Frequentist probability and frequentist statistics. Synthese.

[38] Pearson K. (1900). X. On the criterion that a given system of deviations from the probable in the case of a correlated system of variables is such that it can be reasonably supposed to have arisen from random sampling. The London, Edinburgh, and Dublin philosophical magazine and journal of. Science.

[39] Pearson K. (1906). Note on the significant or non-significant character of a sub-sample drawn from a sample. Biometrika.

[40] Pegler S., Underhill J. (2010). Evaluating the safety and effectiveness of new drugs. Am Fam Physician.

[41] Rafi Z., Greenland S. (2020). Semantic and cognitive tools to aid statistical science: replace confidence and significance by compatibility and surprise. BMC Med Res Methodol.

[42] Rovetta A. (2024). Multiple confidence intervals and Surprisal intervals to avoid significance fallacy. Cureus.

[43] Rovetta A. (2024). S-values and Surprisal intervals to replace P-values and confidence intervals: Accepted - January 2024. Rev Stat J.

[44] Rovetta A. (2024). Statistical significance misuse in public health research: an investigation of the current situation and possible solutions. J Health Policy Outcomes Res.

[45] Rubin M. (2020). “Repeated sampling from the same population?” a critique of Neyman and Pearson’s responses to Fisher. Eur J Philos Sci.

[46] Rubin M. (2021). What type of Type I error? Contrasting the Neyman–Pearson and Fisherian approaches in the context of exact and direct replications. Synthese.

[47] Thiese M.S., Arnold Z.C., Walker S.D. (2015). The misuse and abuse of statistics in biomedical research. Biochem Med.

[48] Ting C., Greenland S. (2024). Forcing a deterministic frame on probabilistic phenomena: a communication blind spot in media coverage of the “replication crisis”. Sci Commun.

[49] Uygun Tunç D., Tunç M.N., Lakens D. (2023). The epistemic and pragmatic function of dichotomous claims based on statistical hypothesis tests. Theory Psychol.

[50] Wasserstein R.L., Lazar N.A. (2016). The ASA statement on p-values: context, process, and purpose. Am Stat.

[51] Yeh R.W., Valsdottir L.R., Yeh M.W., Shen C., Kramer D.B., Strom J.B. (2018). Parachute use to prevent death and major trauma when jumping from aircraft: randomized controlled trial. BMJ (Clin Res).

[52] Good I.J. (1952). Rational decisions. J R Stat Soc [Ser B].

